# Acoustic cardiac triggering: a practical solution for synchronization and gating of cardiovascular magnetic resonance at 7 Tesla

**DOI:** 10.1186/1532-429X-12-67

**Published:** 2010-11-16

**Authors:** Tobias Frauenrath, Fabian Hezel, Wolfgang Renz, Thibaut de Geyer d'Orth, Matthias Dieringer, Florian von Knobelsdorff-Brenkenhoff, Marcel Prothmann, Jeanette Schulz Menger, Thoralf Niendorf

**Affiliations:** 1 Berlin Ultrahigh Field Facility (B.U.F.F.), Max-Delbrueck Center for Molecular Medicine, Berlin, Germany; 2 Siemens Healthcare, Erlangen, Germany; 3 Working Group on Cardiovascular Magnetic Resonance, Medical University Berlin, Charité Campus Buch, HELIOS-Klinikum Berlin-Buch, Dept. of Cardiology and Nephrology, Berlin, Germany; 4 Experimental and Clinical Research Center, Charité - Campus Buch, Humboldt-University, Berlin, Germany

## Abstract

**Background:**

To demonstrate the applicability of acoustic cardiac triggering (ACT) for imaging of the heart at ultrahigh magnetic fields (7.0 T) by comparing phonocardiogram, conventional vector electrocardiogram (ECG) and traditional pulse oximetry (POX) triggered 2D CINE acquisitions together with (i) a qualitative image quality analysis, (ii) an assessment of the left ventricular function parameter and (iii) an examination of trigger reliability and trigger detection variance derived from the signal waveforms.

**Results:**

ECG was susceptible to severe distortions at 7.0 T. POX and ACT provided waveforms free of interferences from electromagnetic fields or from magneto-hydrodynamic effects. Frequent R-wave mis-registration occurred in ECG-triggered acquisitions with a failure rate of up to 30% resulting in cardiac motion induced artifacts. ACT and POX triggering produced images free of cardiac motion artefacts. ECG showed a severe jitter in the R-wave detection. POX also showed a trigger jitter of approximately Δt = 72 ms which is equivalent to two cardiac phases. ACT showed a jitter of approximately Δt = 5 ms only. ECG waveforms revealed a standard deviation for the cardiac trigger offset larger than that observed for ACT or POX waveforms.

Image quality assessment showed that ACT substantially improved image quality as compared to ECG (image quality score at end-diastole: ECG = 1.7 ± 0.5, ACT = 2.4 ± 0.5, p = 0.04) while the comparison between ECG vs. POX gated acquisitions showed no significant differences in image quality (image quality score: ECG = 1.7 ± 0.5, POX = 2.0 ± 0.5, p = 0.34).

**Conclusions:**

The applicability of acoustic triggering for cardiac CINE imaging at 7.0 T was demonstrated. ACT's trigger reliability and fidelity are superior to that of ECG and POX. ACT promises to be beneficial for cardiovascular magnetic resonance at ultra-high field strengths including 7.0 T.

## Background

The challenge of synchronization of data acquisition with the cardiac cycle constitutes a practical impediment of cardiovascular magnetic resonance (CMR). Cardiac motion has been addressed by synchronization strategies exploiting (i) finger plethysmography[1], (ii) cardiac activity related esophageal wall motion[2], (iii) invasive left ventricular blood pressure gating[3], (iv) Doppler ultrasound[4], (v) motion induced changes in the impedance match of RF-coils[5], (vi) self gating techniques[6-10] and optic acoustic methods [11] including human and animal studies. In current clinical CMR, cardiac motion is commonly dealt with using electrocardiographic (ECG) or finger pulse oximetry (POX) triggering/gating techniques [12-14] to synchronize data acquisition with the cardiac cycle. At higher magnetic field strengths the artifact sensitivity of ECG recordings and even of advanced vector ECG increases [13,15]. ECG, being an inherently electrical measurement with electrically active components [12], does carry a risk of surface heating of patients' skin and even of skin burns resulting from induction of high voltages in ECG hardware [16-19]. As ultrahigh field CMR becomes more widespread, the propensity of ECG recordings to interference from electromagnetic fields and to magneto-hydrodynamic effects is further pronounced [20-22].

Realizing the constraints of conventional ECG, a MR-stethoscope which uses the phonocardiogram has been proposed. Its feasibility for the pursuit of/prospectively triggered and retrospectively gated cardiac imaging has been demonstrated for field strengths up to 3.0 T [20,21,23] The applicability and clinical efficacy of acoustic cardiac triggering (ACT) has not been demonstrated for imaging of the heart at ultrahigh magnetic fields yet due to the lack of appropriate RF coils and other practical obstacles of CMR at 7.0 T. Recently, R-wave mis-registration has been consistently reported for ECG triggered CMR at 7.0 T [24-26]. Consequently, in one study approximately 20% of the healthy subjects needed to be excluded from left ventricular function assessment [24,25]. In another study 80% of the acquisitions were gated using pulse oximetry due to ECG-triggering problems [26]. Driven by the limitations and motivated by the challenges of conventional ECG together with the advantages of ACT, this study compares phonocardiogram triggered, conventional vector electrocardiogram triggered and traditional pulse oximetry triggered CMR cine imaging at 7.0 T in a pilot study as precursor to a larger clinical study.

To accomplish this goal, the suitability, accuracy and reproducibility of each cardiac triggering approach for the assessment of left ventricular parameter at 7.0 T is explored. For this purpose, breath-held 2D CINE imaging in conjunction with a retrospective triggering regime is conducted paralleled by real time logging of the ECG, POX and ACT signal waveforms to track (mis)synchronization between the cardiac cycle and data acquisition. A qualitative and quantitative analysis of 2D CINE images and signal waveforms is performed. The merits and limitations of the acoustic cardiac gating approach are discussed and its implications for other ultrahigh field MR imaging applications are considered.

## Materials and methods

### Acoustic Noise Measurements at 7.0 T

During CMR, recordings of a phonocardiogram inside of the magnet bore are paralleled by acoustic noise due to gradient coil switching consisting of several very sharp harmonic components, which are related to the echo time TE and the repetition time TR. For this reason, acoustic measurements were conducted to assess the acoustic signal-to-noise ratio between the sound pressure level induced by the cardiac activity and the sound-pressure level generated by the gradient noise. Acoustic measurements were conducted inside the 7.0 T magnet bore. Two series of acoustic signals were acquired. The first series was designed to record and characterize the noise generated in the MR environment by a 2D CINE FLASH sequence (TE = 2 ms, TR = 4 ms, pixel bandwidth = 445 Hz, FOV = (32 × 32) cm^2 ^using an optical microphone (MO 2000 set, Sennheiser, Wedemark, Germany). For this purpose, the optical microphone was positioned at the same position with respect to the scanning table and the magnet bore as it was used in the volunteer study. The second series was setup to collect and analyze the phonocardiogram derived from the heart sound of healthy subjects superimposed by the environmental noise generated by the same 2D CINE FLASH technique. For this purpose, an acoustic sensor (diameter = 5 cm) covered by a membrane to generate a pressure wave in a waveguide was attached to the optical microphone. The acoustic signal was recorded by means of an USB-soundcard chip (PCM2903; Texas Instruments, Dallas, TX, USA). The optical microphone was calibrated with a 94 dB test tone provided from a pistonphone (Voltcraft SLC-100, Conrad electronics, Germany). After connecting the membrane to the microphone the set-up was calibrated manually to correct for the minor attenuation induced by the acoustic sensor's membrane. With this set-up absolute sound pressure levels were obtained. Furthermore, this approach offers the benefit that the characteristics of the acoustic signal measurements are given by the environment and not by the signal processing unit. Due to the use of this linear time invariant network approach frequency-shifts between the acoustic signal obtained from the MR environment and the final power spectrum can be avoided. Also, the sound pressure level can be normalized to the auditory threshold.

### Pulse Oximetry, ECG and Acoustic Triggering

For pulse oximetry, a commercial sensor (Siemens, Erlangen, Germany) was placed on the tip of the right index finger to track changes in the absorbance due to the pulsing arterial blood. A wireless connection linked the sensors output to the internal physiological signal controller circuitry of the MR scanner.

For ECG recording and triggering, a commercial vector ECG module (Siemens, Erlangen, Germany) was used. The electrodes (ConMed, Corp., Utica, NY, USA) of the vector ECG were carefully placed at the anterior chest wall, with one electrode on the sternum, one on the left thorax, and one below the sternum following the manufacturer's patient preparation instructions. For the vector ECG's training period the patient table was placed in the home position to eliminate major interferences with the fringe field. The waveform was delivered to the internal physiological signal controller circuitry of the clinical MR scanner.

Unlike traditional ECG-triggering the acoustic approach employs the phonocardiogram's first heart tone for triggering instead of electrophysiological signals [23]. The acoustic gating device comprises four main components: an acoustic sensor made of synthetic material placed on the subject's anterior chest for phonocardiogram detection, an acoustic wave guide for signal transfer and to ensure galvanic decoupling, a signal processing unit and a coupler unit to the MR system. The two former aspects have also safety implications since the ACT approach galvanically isolates the subject and hence eliminates the risk for patient burns. Signal processing and conversion were conducted outside the magnet room using dedicated electronic circuit [23]. Short rectangular shaped trigger pulses were generated to provide an output trigger signal. This waveform was delivered to the internal physiological signal controller circuitry of the clinical MR scanner. This design was chosen to meet the needs of cardiac gated/triggered CMR: (i) maximum latency of 35 ms between the ECG's R-wave and phonocardiogram based trigger output pulse, (ii) free of interference with electromagnetic fields and (iii) immunity to magneto-hydrodynamic effects. The current implementation connects the trigger signal to the MR-scanner's standard external trigger signal input. Hence, no changes to the MR system's hardware or software are required [23]. The acoustic sensor was positioned directly on the subject's chest at the anterior left fifth intercostals space and gently fixed with a strap incorporated in the MR patient table. Note that neither the limited ECG in the MR scanner nor the acoustic waveforms reported here should be treated as reliable indicators of patient emergency conditions.

Three patient table positions were selected to examine the signal waveform as a function of the magnetic field strength: (i) patient table in home position so that the magnetic field strength at the ECG, POX and ACT sensor position was approximately 0.3 T, (ii) ECG, POX and ACT sensor position aligned with the front end of the magnet with a fringe field of approximately 1.0 T and (iii) ECG, POX and ACT sensor in the magnet's isocenter (B_0 _= 7.0 T).

### Logging and Analysis of Signal Waveforms at 7.0 T

For all subjects vector ECG, POX and ACT were connected at the same time to record traces of waveforms along with the trigger information simultaneously. This information was extracted from the scanners central physiological monitoring unit (CPMU) and simultaneously stored in log files with a sampling rate of 400 Hz (ECG), 50 Hz (POX) and 200 Hz (ACT). Also, the trigger detection tickmarks generated for Vector ECG, POX or ACT triggering by the scanners CPMU were written into log files. This logging procedure was paralleled by storing the respiratory trace simultaneously using a sampling rate of 50 Hz.

The recorded data were processed to analyze the trigger information and to assess triggering efficiency and temporal fidelity of synchronization with the cardiac cycle for each trigger technique. Off-line analysis of the log-files was performed using LabVIEW (National Instruments, Austin, TX, USA). For this purpose a customized post-processing algorithm was developed. The post-processing procedure includes the following features:

• Identification of breath hold periods: Only portions of the trigger signal traces which were acquired during breath-held 2D CINE FLASH imaging were included into the waveform analysis. For this purpose, the respiratory trace was used.

• Segmentation and temporal realignment: The ECG waveform is segmented into individual R-R intervals by using cross correlation between R-R intervals. For this purpose, one R-R interval acquired at the one breath-held CINE series is taken as a reference while all other R-R intervals are shifted along the time axis to achieve maximum correlation with the reference. This R-R wave segmentation mask is applied to the segmentation of the POX and ACT waveforms, which were acquired simultaneously to the ECG waveform. Temporal realignment was used to overcome the potential bias of (erroneous) trigger detection provided by the scanners internal real-time circuitry.

• Reassignment of the trigger detection tickmarks derived from the scanners central physiological monitoring unit to the realigned ECG, POX and ACT traces and assessment of the trigger jitter across the cardiac cycle.

• Calculation of the mean value and the standard deviation of the cardiac cycle for ECG, POX and ACT as an objective measure for trigger reliability.

• Calculation of the offset between the ECG's R-wave and the trigger detection moment derived from the ECG, POX and ACT waveform as an objective measure for trigger reliability.

### Cine CMR at 7.0 T

End-expiratory breath-hold short axis views of the heart ranging from the atrioventricular ring to the apex were acquired using retrospectively gated 2D FLASH CINE on a 7.0 T whole body MR systems (Magnetom, Siemens, Erlangen, Germany). A 4 element transmit/receive coil was used for RF excitation and signal reception [27]. The coil was connected to the 7.0 T system via 4 transmit/receive (T/R) switches and a 1 to 4 radio frequency power splitter and combiner with a CP-like phase setting for the four individual channels. The coil setup consists of two identical coil subsets - one placed on the subject's anterior torso and one positioned posterior - each containing two transmit/receive loop coil elements.

For breath-held 2D CINE FLASH imaging field of view (FOV) was set to (340 × 308) mm^2^, data acquisition matrix size was set to 256 × 186 elements (reconstruction matrix size 256 × 232 elements). 30 cardiac phases (temporal resolution = 33 ms for a heart rate of 60 bpm) were acquired using typically 18 slices (slice thickness = 4 mm, slice gap = 2 mm). Slice order was reversed from apex-base to base-apex throughout the set of subjects to eliminate patient discomfort or training effects. Image acquisition was confined to a single slice per breath-hold. The flip angle was set to α = 35 for all subjects, resulting in TR = 5.5 ms and TE = 2.7 ms. Parallel imaging was applied (R = 2) using sensitivity encoding based reconstruction.

Three sets of breath-held 2D CINE FLASH acquisitions were performed. In one set ACT was employed. For comparison, the other set made use of vector ECG based cardiac triggering while another set used POX for cardiac triggering. The use of ACT, ECG and POX was swapped randomly to avoid systematic errors.

### Image Analysis

For LV chamber quantification end-diastolic and end-systolic volume (EDV, ESV), and left ventricular mass (LVM) were calculated using commercial evaluation software (CMR42^®^, Circle Cardiovascular Imaging, Calgary, Canada) from images of all subjects using all three triggering methods. CMR reading was performed by one cardiologist with very profound expertise in clinical CMR (>3000 CMR examinations), who was not involved in the image acquisition at all.

For CINE image quality assessment two independent observers reviewed and scored the images in a randomized, blinded reading session. For this purpose, overall image quality of end-diastolic and end-systolic images was rated using a scale ranging from 0 to 3 for each slice. The following scale was used for the blinded reading:

0 - images with poor and non-diagnostic quality due to cardiac motion induced blurring,

1 - image quality impaired by cardiac motion which may lead to misdiagnosis,

2 - good image quality, cardiac motion artifacts hard to recognize and

3 - excellent image quality, no cardiac motion artifacts observed.

After the independent image quality assessment was completed both readers exchanged their rankings for each case-, slice- and cardiac phase, and agreed on a consensus score.

Endocardial border sharpness (EBS) of the 2D CINE FLASH images derived from ACT, ECG POX triggered acquisitions was determined through an objective measurement of acutance using a dedicated algorithm [21].

### Study Population

The study was designed as a comparative volunteer study using healthy adult subjects with no history of cardiovascular disease (n = 9). The mean age was 32 ± 10 years ranging 23-52 years. The average body surface area was (2.0 ± 0.3) m^2 ^ranging from 1.8 m^2 ^to 2.7 m^2^. The average body mass index was 24.8 ± 4.4 kg/m^2 ^(range 20.9-35.9 kg/m^2^). Volunteers with contraindications to CMR were excluded. The study was carried out according to the principles of the Declaration of Helsinki and was approved by the local institutional ethics committee. Informed written consent was obtained from each volunteer prior to the study, in compliance with the local institutional review board guidelines.

### Statistical Analysis

All data are presented as mean ± standard deviation (SD) unless stated otherwise. Statistical significance in the difference of the image scores was analyzed using Wilcoxon matched pairs test for the consensus scores for ACT vs ECG, ACT vs POX and ECG vs POX triggered data. Statistical significance in the difference of the parameter derived from LV function assessment was analyzed using t-test. A probability p ≤ 0.05 was considered statistically significant. All computations were performed with Microsoft Excel (Microsoft, Redmond, USA) and R (R Foundation for Statistical Computing, Vienna, Austria). Comparison of the different triggering techniques was carried out using the Bland and Altman method [28] (GraphPad Software, Inc., La Jolla, CA, USA). The confidence interval was set to the mean value ± 1.96 of the standard deviation.

## Results

### Acoustic Signal-to-Noise Measurements at 7.0 T

**Figure 1:**
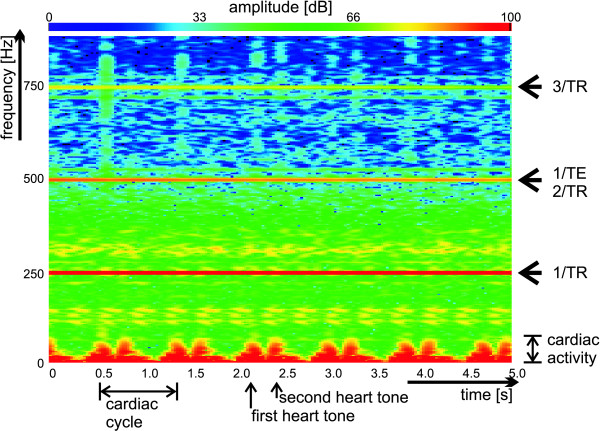
**Acoustic Spectrogram obtained at 7.0 T**. Spectrogram obtained from a subject positioned at the 7.0 T magnet's isocenter during 2D CINE FLASH acquisitions (TE = 2.0 ms, TR = 4.0 ms). The graphs show signal contributions from gradient switching superimposed on the cardiac signals. The gradient switching manifests itself by several very sharp harmonic components at 1/TR, 2/TR, 3/TR and 1/TE with maximum sound pressure level close to 120 dB. The spectrogram also shows the 1^st ^and the 2^nd ^heart tone which are of low-frequency nature. Acoustic signal-to-noise ratio, which is defined as the ratio between the sound pressure level due to cardiac activity and the gradient switching induced sound pressure level is approximately 30 dB for the frequency range between 10 Hz and 70 Hz.

Recordings of phonocardiograms inside the magnet bore are paralleled by acoustic noise due to gradient coil switching. High sound pressure levels (SPL) of up to 120 dB were induced by magnetic field gradients driven by the switching scheme of a 2D FLASH CINE imaging technique. Figure 1 illustrates the spectrogram which shows the sound pressure level spectrum over time obtained from a subject positioned at the magnet's isocenter during 2D CINE FLASH imaging (TE = 2.0 ms, TR = 4.0 ms) at 7.0 T. In this case of the phonocardiogram being paralleled by the gradient switching noise the sound pressure level (SPL) was tracked over a series of 6 heartbeats. The power spectra show numerous noise peaks including several very sharp harmonic components at 1/TR, 2/TR, 3/TR and 1/TE which are related to the gradient switching scheme of 2D FLASH with maximum SPL close to 120 dB. The spectrogram also revealed that the heart sound encompasses low-frequencies.

SNR was defined as the ratio between the sound pressure level due to cardiac activity and the gradient switching induced sound pressure level. For example, for the frequency range between 10 Hz and 50 Hz a minimum signal to noise ratio of SNR = 30 dB was found for 2D CINE FLASH imaging at 7.0 T. To make acoustic triggering immune to interference from acoustic noise generated by gradient switching, separation of the acoustic cardiac activity from the higher frequency gradient noise was carried out by means of a third order inverse Chebychev filter using a UAF42 chip (Burr Brown Products by TI, Dallas, Texas, USA). The cut off frequency was set to f_c _= 105 Hz since the energy of the first heart tone is mainly covered by frequencies ranging from 1 to 100 Hz. With this filtering no gradient noise peaks or peaks due to environmental noise were found within the relevant frequency range between 0 Hz to 105 Hz. The filtering yielded an attenuation of the gradient noise peaks found for frequencies above the cut-off frequency f_c _= 105 Hz by at least 30 dB.

### Assessment of ECG, POX and ACT Waveforms at 7.0 T

**Figure 2:**
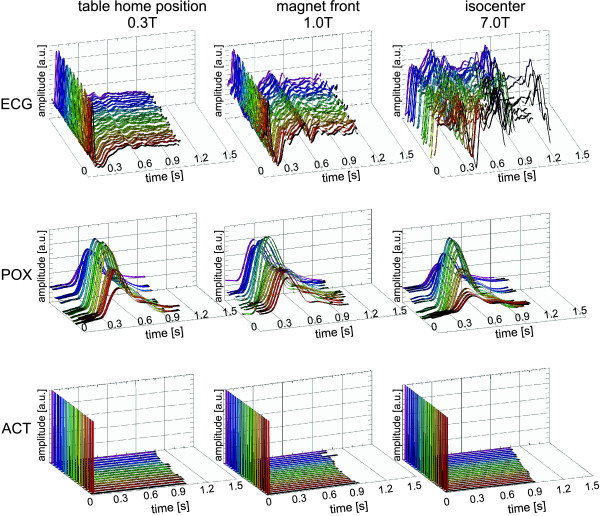
**ECG, pulse oximetry and acoustic trigger signal traces acquired at three different field strengths**. ECG **(top)**, pulse oximetry **(middle) **and acoustic trigger **(bottom) **signal traces derived from a healthy subject. Signal traces were recorded with the ECG, ACT and POX sensors located at the patient table home position **(left)**, at the front end of the 7.0 T magnet **(center) **and in the isocenter of the 7.0 T magnet **(right) **and post-processed and filtered by the scanners central physiological monitoring unit. Severe signal distortion occurred in the vector ECG signal obtained at the magnet's isocenter. A scatter in the amplitude and width was observed for the peak in the pulse oximetry trace. ACT is free of interferences with electromagnetic fields and magneto-hydrodynamic effects.

Off-line analysis of the log-files revealed that ECG waveforms were susceptible to severe distortions. Adverse signal elevation was found for cardiac phases where normally the T-wave occurs; a magneto-hydrodynamic effect which was pronounced at the isocenter of the magnet as illustrated in Figure 2, which shows ECG, POX and ACT traces derived from an individual subject over 18 cardiac cycles. ECG waveform distortions yielded an amplitude reaching the same order of magnitude or even larger than that of the R-wave. Pulse oximetry waveforms were free of interference from electromagnetic fields and magneto-hydrodynamic effects. A rather flat plateau and a significant scatter in the amplitude and width were observed for the peak in the POX trace. The peak in the POX trace showed a mean latency of approximately 350 ms with respect to the R-wave of the ECG trace. The acoustic approach provided waveforms free of interferences from electromagnetic fields or from magneto-hydrodynamic effects even in the isocenter of the 7.0 T magnet as illustrated in Figure 2. Off-line analysis of ECG and ACT traces yielded an average delay of Δt = 29.65 ms ± 4.43 ms between the R-wave and the first heart tone. This delay is not detrimental for whole R-R coverage 2D CINE FLASH using retrospective triggering.

### Cardiac 2D CINE FLASH Imaging at 7.0 T

**Figure 3:**
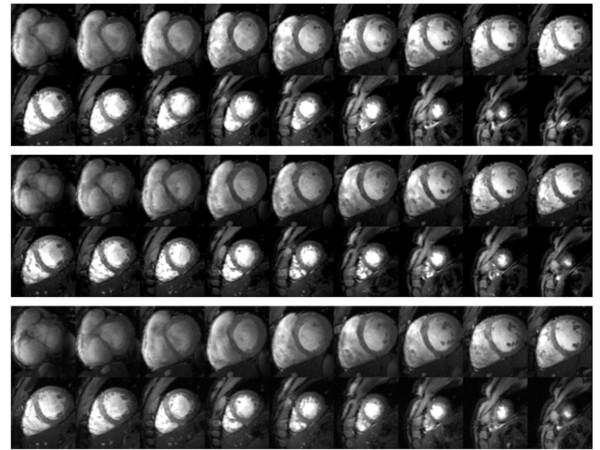
**Short axis diastolic views free of cardiac motion effects**. Short axis diastolic views obtained from breath-held base-to-apex 2D CINE FLASH acquisitions using **(top) **vector ECG, **(middle) **ACT and POX **(bottom) **gating. In this case of correct R-wave detection (subject 1), ECG-gated 2D CINE FLASH imaging was found to be immune to cardiac motion effects, as were ACT and POX gated acquisitions.

**Figure 4:**
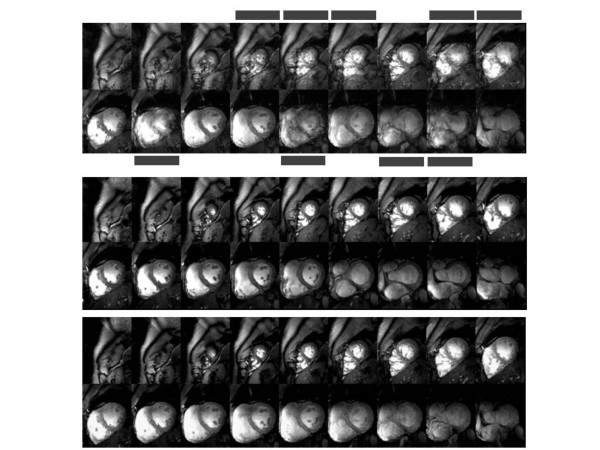
**Short axis diastolic views showing cardiac motion artifacts when using ECG gating**. Short axis diastolic views obtained from breath-held apex-to-base 2D CINE FLASH acquisitions using **(top) **vector ECG, **(middle) **ACT and POX **(bottom) **gating. In this subject (subject 2) vector ECG triggered 2D CINE FLASH imaging was prone to severe cardiac motion artifacts if R-wave mis-registration occurred. Images suffering from cardiac motion induced blurring are marked with grey bars. Acoustic triggering and POX provided image quality free of interferences from cardiac motion effects.

In the case of correct R-wave detection, ECG-gated 2D CINE FLASH imaging was found to be immune to cardiac motion effects as illustrated in figure 3 for one of the 9 subjects (subject 1). However, frequent R-wave mis-registration occurred in ECG-triggered acquisitions with a failure rate of up to 30% which manifests itself in a severe jitter of the R-wave detection tickmarks. Consequently, ECG triggered 2D CINE FLASH imaging was prone to severe cardiac motion artifacts if R-wave mis-registration occurred. For example, an ECG cardiac triggered whole heart coverage 2D CINE FLASH dataset obtained at diastole is shown in the top row of Figure 4 for one subject of the 9 subjects (subject 2). Images suffering from cardiac motion induced blurring are marked with dotted lines. Unlike ECG, ACT triggering produced images free of cardiac motion artefacts as illustrated in the bottom row of Figure 4 for the same subject. POX triggering 2D CINE FLASH acquisitions obtained from the same subject also produced images free of blood pulsation and cardiac motion artefacts as demonstrated in the middle row of Figure 4.

### Assessment of the Trigger Detection Variance

**Figure 5:**
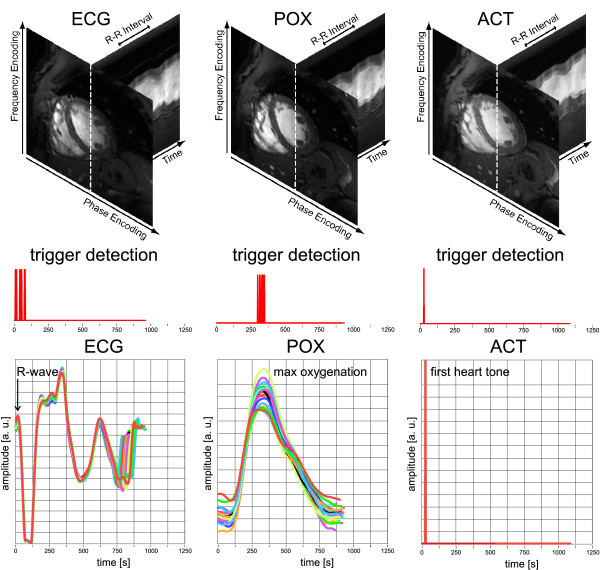
**Example of correct ECG trigger detection**. Cardiac images, trigger detection tickmarks and signal waveforms obtained at 7.0 T using vector ECG (left), pulse oximetry (center) and acoustically triggered (right) 2D CINE FLASH acquisitions. All signal waveforms show the output of the scanners central physiological monitoring unit (including processing of the ECG, POX and ACT signal) as displayed at the scanners user interface. Top: Mid-ventricular, short axis views of the heart together with whole R-R interval time series of one-dimensional projections along the profile (dotted line) marked in the short axis view. Middle: Trigger detection tickmarks obtained from a single subject over 18 cardiac cycles after temporal realignment using cross correlation and reassignment. Bottom: Signal waveforms obtained from a single subject (subject 1) over 18 cardiac cycles. In spite of vector ECG's severe signal distortion faultless vector ECG triggering was observed for this example. Hence, in this example of correct recognition of the onset of cardiac activity, vector ECG, POX and ACT triggered 2D CINE FLASH imaging were found to be immune to the effects of cardiac motion. Please note the jitter in the vector ECG (Δt = 60 ms) and in the pulse oximetry trigger (Δt = 65 ms)detection.

Figure 5 shows mid-ventricular short axis views of the heart together with whole R-R interval time series of one-dimensional projections, trigger detection tickmarks and signal waveforms obtained at 7.0 T using ECG, POX and ACT triggered 2D CINE FLASH acquisitions for a midventricular slice derived from subject 1. In this example of almost correct recognition of the onset of cardiac activity, ECG, POX and ACT triggered 2D CINE FLASH imaging were found to be rather immune to the effects of cardiac motion. Consequently, the 2D CINE FLASH images derived from ECG, ACT and POX acquisitions together with the M-mode like whole R-R interval time series of one-dimensional projections along the cardiac phases safeguard recognition and delineation of the ventricular blood/myocardium interface. In spite of ECG's severe signal distortion faultless ECG triggering was observed for this example, with the exception of a scatter in the ECG trigger detection of approximately Δt = 60 ms which might compromise the temporal fidelity and hence might constitute a synchronization problem. Please note that a variance was also observed for the peak amplitude and peak width of the POX waveform as pointed out in Figure 5. This variance resulted in a trigger detection jitter of approximately Δt = 65 ms, which is equivalent to two cardiac phases. In comparison, ACT showed a 5 ms jitter which can be attributed to the ACT waveform sampling rate of 200 Hz.

**Figure 6:**
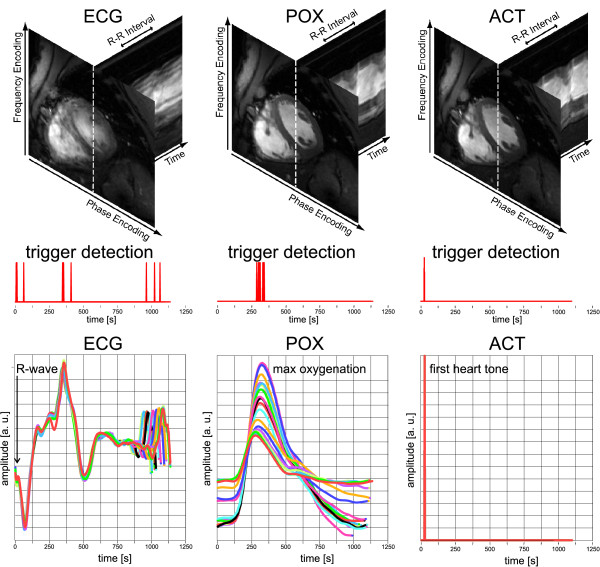
**Example of erroneous ECG trigger detection**. Cardiac images, trigger detection tickmarks and signal waveforms obtained at 7.0 T using vector ECG **(left)**, pulse oximetry **(center) **and acoustically triggered **(right) **2D CINE FLASH acquisitions. All signal waveforms show the output of the scanners central physiological monitoring unit (including processing of the ECG, POX and ACT signal) as displayed at the scanners user interface. **Top**: Mid-ventricular, short axis views of the heart together with whole R-R interval time series of one-dimensional projections along the profile (dotted line) marked in the short axis view. **Middle**: Trigger detection tickmarks obtained from a single subject over 18 cardiac cycles after temporal realignment using cross correlation and reassignment. **Bottom**: Signal waveforms obtained from a single subject (subject 2) over 18 cardiac cycles. In this example vector ECG triggered CINE imaging was prone to severe cardiac motion artifacts due to R wave mis-registration which induced reduction in myocardium/blood contrast and image sharpness. ACT triggered 2D CINE FLASH imaging provided faultless trigger detection and hence produced images free of motion artifacts. Please note the scatter in the POX peak amplitude and peak width, causing a jitter (Δt = 72 ms) in the pulse oximetry trigger detection which constituted a synchronization problem.

Figure 6 shows an example of erroneous ECG triggering for a midventricular slice derived from subject 2. In this case, ECG triggered 2D CINE FLASH imaging was prone to severe cardiac motion artifacts due to R wave mis-registration. Trigger detection was found to be scattered across several cardiac phases including early systole and diastole as demonstrated by the tickmarks depicted in Figure 6. R-wave mis-registration induced reduction in myocardium/blood contrast and image sharpness as illustrated by the short axis views together with whole R-R interval time series of one-dimensional projections. In comparison, ACT triggered 2D CINE FLASH imaging provided faultless trigger detection, accurate to the peak induced by the 1^st ^heart tone and hence produced images free of motion artifacts. Please note the scatter in the POX peak amplitude and peak width, causing a jitter (Δt = 72 ms) in the pulse-oximetry trigger detection.

Mean R-R interval lengths deduced from the signal waveforms of ECG, POX and ACT triggered acquisitions are surveyed for each subject in Table 1 together with the standard deviation of the R-R interval length, which is a measure of the trigger detection accuracy. A close match in the mean R-R interval length and in the standard deviation of the R-R interval length derived from the assessment of the ECG, ACT and POX waveforms signifies correct trigger detection. A significant difference in the standard deviation of the R-R interval length deduced from ECG, ACT and POX waveforms indicates trigger (mis)registration at cardiac phases other than that which mark the onset of cardiac activity. Four out of nine healthy subjects showed a standard deviation for the cardiac cycle derived from ECG waveforms which was at least 1.5 times larger (SD_ECG _≥ 1.5 * SD_ACT_) than that obtained from ACT or POX waveforms.

**Table 1:** Synopsis of the trigger detection variance assessment

	Mean cardiac interval length			Standard deviation of the mean cardiac interval length			Standard deviation of the cardiac trigger offset		
Subject	(ms)			(ms)			(ms)		
	ACT	ECG	POX	ACT	ECG	POX	ACT	ECG	POX
1	869	865	840	89	54	45	78	10	11
2	870	795	1103	83	126	87	76	477	101
3	875	845	834	48	28	34	13	3	19
4	1064	999	1079	76	143	52	44	456	456
5	926	990	923	49	54	31	14	56	23
6	841	939	898	95	69	35	149	52	15
7	848	839	876	79	62	48	75	257	67
8	1110	886	1091	37	174	33	1	1047	14
9	762	576	660	81	168	144	112	488	331

Synopsis of the mean R-R interval lengths, standard deviation of the mean R-R interval lengths and standard deviation of the cardiac trigger detection offset deduced from the signal waveforms of ECG, POX and ACT triggered acquisitions. A significant difference in the standard deviation of the R-R interval length deduced from ECG, ACT and POX waveforms indicates trigger misregistration. A large standard deviation of the offset between the ECG's R-wave and the trigger detection which was derived from the ECG, POX and ACT waveforms indicates trigger misregistration

Table 1 also surveys the standard deviation of the offset between the ECG's R-wave and the trigger detection moment which was derived from the ECG, POX and ACT waveforms and which was applied for synchronization. Four out of nine healthy subjects showed a standard deviation for the cardiac trigger offset derived from ECG waveforms which was at least four times larger (SD_ECG _≥ 4 * SD_ACT_) than that observed for ACT or POX waveforms.

### Left Ventricular Parameter Assessment

**Figure 7:**
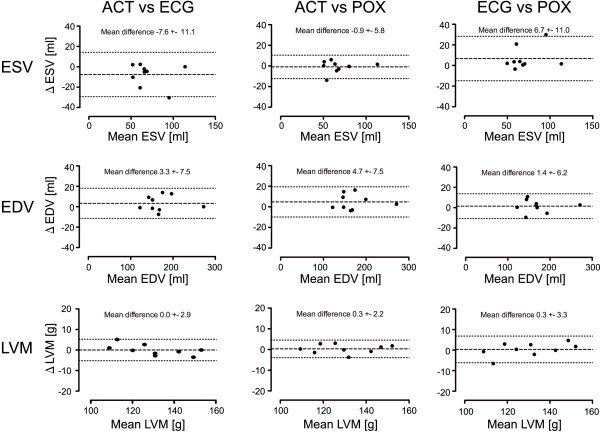
**Comparison of cardiac chamber quantification parameters obtained for three triggering methods**. Bland-Altman plots showing for each subject the mean of two measurements (ACT vs ECG, ACT vs POX and ECG vs POX) and the difference in the left ventricular parameter derived from vector ECG, ACT and POX gated 2D CINE FLASH acquisitions at 7.0 T. Dashed black lines in the Bland-Altman plots represent the mean difference while the dotted lines embody the confidence interval which was set to the mean value ± 1.96 of the standard.

Left ventricular volumes, mass and ejection fraction deduced from ECG, POX and ACT triggered acquisitions are surveyed in Figure 7. For each subject, the mean of the two measurements (ACT vs. ECG and ACT vs. POX) and the difference between the LV parameter obtained for ECG, POX and ACT triggered 2D CINE FLASH acquisitions are shown using Bland-Altman plots. The mean LV parameter derived from ECG triggered 2D CINE FLASH acquisitions were ESV = 74 ± 22ml, EDV = 170 ± 43ml, LVM = 130 ± 17) g, EF = 57 ± 8%. The mean LV parameter obtained from POX triggered 2D CINE FLASH acquisitions showed ESV = 67 ± 19 ml, EDV = 168 ± 44 ml, LVM = 130 ± 15g, EF = 60 ± 5%. In comparison, the ACT-triggered acquisitions yielded: ESV = 66 ± 20 ml, EDV = 173 ± 43 ml, LVM = 130 ± 15g, EF = 62 ± 3%. T-test revealed no significant differences for LV parameter derived from ACT, POX and ECG triggered acquisitions (all p > 0.05).

### Image Quality Assessment

The application of acoustic triggering substantially improved the 2D CINE FLASH image quality as compared to the conventional approach. The blinded reading yielded a mean image quality score of 1.7 ± 0.5 for end-diastolic and 1.3 ± 0.6 for end-systolic cardiac images derived from ECG gated acquisitions. In comparison POX gated acquisitions yielded a mean image quality score of 2.0 ± 0.5 for end-diastolic and of 1.6 ± 0.5 for end-systolic cardiac phases. ACT triggered images showed a mean image quality score of 2.4 ± 0.5 for end-diastolic and of 2.0 ± 0.5 end-systolic cardiac phases as summarized in Table 2. A comparison between the image quality score obtained for ACT, POX and ECG gating using Wilcoxon paired test revealed significant differences between ACT vs. ECG gated acquisitions (p = 0.10 for endsystole, p = 0.04 for enddiastole) and for ACT vs. POX gated acquisitions (p = 0.03 for endsystole, p = 0.01 for enddiastole). The image quality comparison between ECG vs. POX gated acquisitions showed no significant differences in image quality (p = 0.40 for endsystole, p = 0.34 for enddiastole).

**Table 2:** Synopsis of the image quality assessment

	End-diastolic Phase			End-systolic Phase		
**Subject**	**ACT**	**ECG**	**POX**	**ACT**	**ECG**	**POX**

1	2,4	2,2	1,8	1,5	1,6	1,7
2	2,6	0,9	1,9	2,1	0,5	1,6
3	2,9	2,7	2,5	2,6	2,4	2,1
4	2,9	1,7	2,8	2,6	1,2	2,4
5	2,5	1,9	1,9	2,1	1,7	1,7
6	1,6	2,1	1,8	1,5	1,5	1,4
7	1,5	1,6	1,1	1,1	1,6	1,0
8	2,8	1,1	2,4	2,4	0,8	1,5
9	2,1	0,7	1,3	1,9	0,5	0,9

**Mean**	**2,4**	**1,7**	**2,0**	**2,0**	**1,3**	**1,6**

**SD **(+/-)	**0,5**	**0,7**	**0,5**	**0,5**	**0,6**	**0,5**

Mean values of image quality scores for each subject and trigger approach. Overall image quality of end-diastolic and end-systolic images was rated using a scale ranging from 0 to 3 for each slice (0 - images with poor and non-diagnostic quality due to cardiac motion induced blurring, 1 - image quality impaired by cardiac motion which may lead to misdiagnosis, 2 - good image quality, cardiac motion artifacts hard to recognize and 3 - excellent image quality, no cardiac motion artifacts observed). Please note that the poor scores obtained for subject 7 are due to respiration induced motion artifacts

In case of faultless gating the EBS analysis revealed similar results for all synchronisation techniques. ECG gated acquisitions showed an average EBS of (2.2 ± 0.3) pixels. ACT gated acquisitions yielded an average EBS of (2. 1 ± 0.2) pixels, and POX gated acquisitions showed an average EBS of (2.1 ± 0.2) pixels. In case of erroneously ECG gated acquisitions EBS analysis was challenging due to heavily reduced contrast to noise ratio (CNR) between blood and myocardium. The CNR degradation was caused by severe signal blurring across the endocardial border, as demonstrated in Figure 4.

## Discussion

CMR at 7.0 T is still in its infancy and needs to continue to be very carefully validated against CMR applications very well established at 1.5 T and 3.0 T [29]. In current basic and clinical research practice some of the traits of ultrahigh field CMR are offset by challenges intrinsic to the use of ultrahigh magnetic field strength such as the synchronization of data acquisition with cardiac motion using traditional electrocardiographic (ECG) techniques. To address this issue, this study examines the applicability of acoustic cardiac triggering for CMR at 7.0 T in healthy volunteers, as a precursor to a larger clinical study. The acoustic approach was found to be immune to interference from environmental and gradient switching induced acoustic fields plus to be free of interference from electromagnetic fields and magneto-hydrodynamic effects. The efficacy and reliability of acoustic triggering is demonstrated by eliminating the frequently-encountered difficulty of mis-triggering due to ECG-waveform distortions or temporal jittering in the pulse-oximetry synchronization. R-wave mis-registration occurred in ECG-triggered acquisitions with a failure rate of up to 30% which manifest itself in severe cardiac motion induced image blurring.

It should be noted, that ECG trigger mis-registration was not equally distributed across the entire cardiac cycle but occurred at cardiac phases with large amplitude or up-slope in the ECG waveform including (i) an initial peak which covers the R-wave and (ii) major waveform distortions at systole and end-diastole. This mis-triggering behavior forces the k-space segmentation and retrospective reconstruction strategy to combine and assign k-space data which were acquired at different phases of myocardial contraction and relaxation to the same cardiac phase used to form a final image. This out-of-sync assignment causes severe degradation in image quality and image sharpness. Please note that this study did not yield missed ECG triggers. Missed triggers would not compromise image quality but lengthen the acquisition time. Acoustically triggered 2D CINE FLASH imaging at 7.0 T produced images free of motion artifacts, as did pulse oximetry triggered 2D CINE FLASH imaging. The latter showed a scatter in the POX peak amplitude and peak width, causing a jitter of approximately Δt = 72 ms in the pulse-oximetry based trigger detection. This might be tolerable for a temporal resolution of 30 ms to 50 ms commonly used in conventional CINE imaging since data acquisition is distributed over several cardiac cycles which might result in averaging of motion effects. However, it stands to reason that the enhanced temporal resolution of CINE imaging facilitated by the signal-to-noise benefit at high and ultra-high fields [30] combined with the speed gain of parallel cardiac imaging [31] can be used to generate highly accurate time-series curves for wall motion tracking (i) to determine the exact time point of maximal systolic contraction and diastolic filling including assessment of mechanical dyssynchrony and (ii) to visualize small rapidly moving structures. Taking the underlying physiological temporal resolution into account it is fair to assume that a jitter in the POX trigger detection larger than 2-3 cardiac phases might diminish the temporal fidelity needed to characterize those physiologic phenomena within the cardiac cycle. Hence, the trigger detection jitter observed for pulse oximetry can constitute a challenge for reliable synchronization of data acquisition with the cardiac cycle. Another drawback of pulse oximetry is the latency between cardiac activity and trigger registration caused by the travel time of blood between the heart and the sensor position which can be in the order of several hundreds of milliseconds but which can also depend on (patho)physiology.

Acoustic triggering presents no risk of high voltage induction and patient burns, patient comfort and ease of clinical use, which all have, patient comfort, safety and practical implications. With reliable ACT triggering available, a positively-inclined practitioner might envisage using the merits of ACT to further simplify clinical CMR. For example, LV assessment is routinely conducted using 2D CINE imaging encompassing a stack of end-expiratory breath-held short axis views of the heart ranging from the atrioventricular ring to the apex. In current clinical practice, it is common to plan and scan each slice in an independent series instead of scanning all slices in a single series because of the frequently encountered risk of mis-triggering. This practical work around bears the advantage that only the most recently acquired slice needs to be re-scanned in case of non-diagnostic image quality due to mis-synchronization. Overcoming the hassle of ECG triggering, reliable triggering using ACT holds the promise to obviate the need for creating an independent series for each slice. Hence ACT, may help to streamline CINE imaging by using a single series for the entire stack of slices which has practical, data storage, data handling and data mining implications.

Admittedly, acoustic cardiac gating shares an apparent drawback of conventional ECG and pulse oximetry based cardiac triggering/gating that extra hardware is required for signal detection and processing, although the current ACT setup does not disturb the scanners certification. To overcome the constraint of using ancillary hardware various self-gating methods have been proposed and it's feasibility has been demonstrated for CMR [7,8,10,32]. However, self-gated CMR of small displacements such as vessel wall motion or MR angiography (MRA) remains a challenge due to the small changes in blood volume, low changes in vessel size and small vessel displacements throughout the cardiac cycle. The acoustic cardiac gating approach reported here is conceptually appealing for the pursuit of vascular CMR since the acoustic sensor can be used to directly detect vessel pulsation from larger vessels included in the target vessel territory.

It should be noted that the work reported here is limited to using vector ECG recordings and retrospective reconstruction techniques implemented on a clinical platform. Further fine-tuning of the post-processing and image reconstruction procedures used in current clinical practice including cross-correlation of ECG signals obtained for different R-R intervals together with retrospective image reconstruction is conceptually appealing to enhance ECG's trigger detection accuracy, albeit this change in the manufacturers ECG processing/reconstruction methodology is beyond the scope of the work reported here.

## Conclusion

The applicability of acoustic triggering for cardiac CINE imaging at 7.0 T was shown. The intrinsic insensitivity of the MR-stethoscope to interference from electro-magnetic fields renders it suitable for left ventricular parameter assessment at 7.0 T due to its excellent trigger reliability, which is superior to that of traditional ECG, VCG and conventional pulse oximetry. Acoustic cardiac triggering promises to be beneficial for ultra-high field strengths including 7.0 T and beyond, which is an important but challenging development looming on the pre-clinical research horizon. Although the full range of ultrahigh field CMR is untapped yet, it is expected to drive future technological developments. With appropriate ancillary triggering hardware, RF-coil design and imaging techniques/protocols customized for 7.0 T applications, LV assessment and other CMR applications are feasible. While this is, for the moment, merely a start, it continues to motivate new basic and clinical research on ultrahigh field CMR, including extra efforts towards the development of a wireless signal transmission version of the acoustic triggering approach.

## Competing interests

Wolfgang Renz is a full-time employee of Siemens (Erlangen, Germany).

## Authors' contributions

TF build the acoustic cardiac triggering device, performed the experiments, collected the data and was significantly involved in writing the manuscript. FH performed data evaluation and statistical analysis and was involved in editing the manuscript.TGO conducted analysis of the waveforms obtained for ACT, ECG and POX and was involved in editing the manuscript. MD has setup the CMR protocols for this study. FvK was responsible for the volunteer care, data documentation, left ventricular function analysis and image quality assessment. He was involved in editing the manuscript. MP performed image quality assessment. He was involved in editing the manuscript. JSM was significantly involved in the study and imaging protocol design. She was involved in editing the manuscript. TN was responsible for the overall concept, was significantly involved in the study and protocol design. He was also significantly involved in writing the manuscript.
